# Supplementary material for: BIAPSS: A Comprehensive Physicochemical Analyzer of Proteins Undergoing Liquid–Liquid Phase Separation
Source: Int J Mol Sci. 2022 May 31;23(11):6204. doi: 10.3390/ijms23116204 (PMC9181037; doi:10.3390/ijms23116204)

Supplementary Materials

BIAPSS: A comprehensive physicochemical analyzer of proteins undergoing liquid-liquid phase separation

Aleksandra E. Badaczewska-Dawid [1,*], Vladimir N. Uversky [2,*] , and Davit A. Potoyan [1,3,4*]

[1] Department of Chemistry, Iowa State University, Ames, IA50011, USA; abadacz@iastate.edu (A.E.B.-D.); potoyan@iastate.edu (D.A.P.)

[2] Department of Molecular Medicine and USF Health Byrd Alzheimer's Research Institute, Morsani College of Medicine, University of South Florida, Tampa, FL 33612, USA; vuversky@usf.edu

[3] Bioinformatics and Computational Biology program, Iowa State University, Ames, IA 50011, USA

[4] Department of Molecular Medicine and USF Health Byrd Alzheimer's Research Institute, Morsani College of Medicine, University of South Florida, Tampa, FL 33612;

[*] Correspondence: abadacz@iastate.edu (A.E.B.-D.), vuversky@usf.edu (V.N.U.); potoyan@iastate.edu (D.A.P.)

The original results generated within BIAPSS web platform for FUS (UniProt identifier: P35637)

1.1 Entry Summary and Annotation web app: *https://biapss.chem.iastate.edu/single_seq.html*

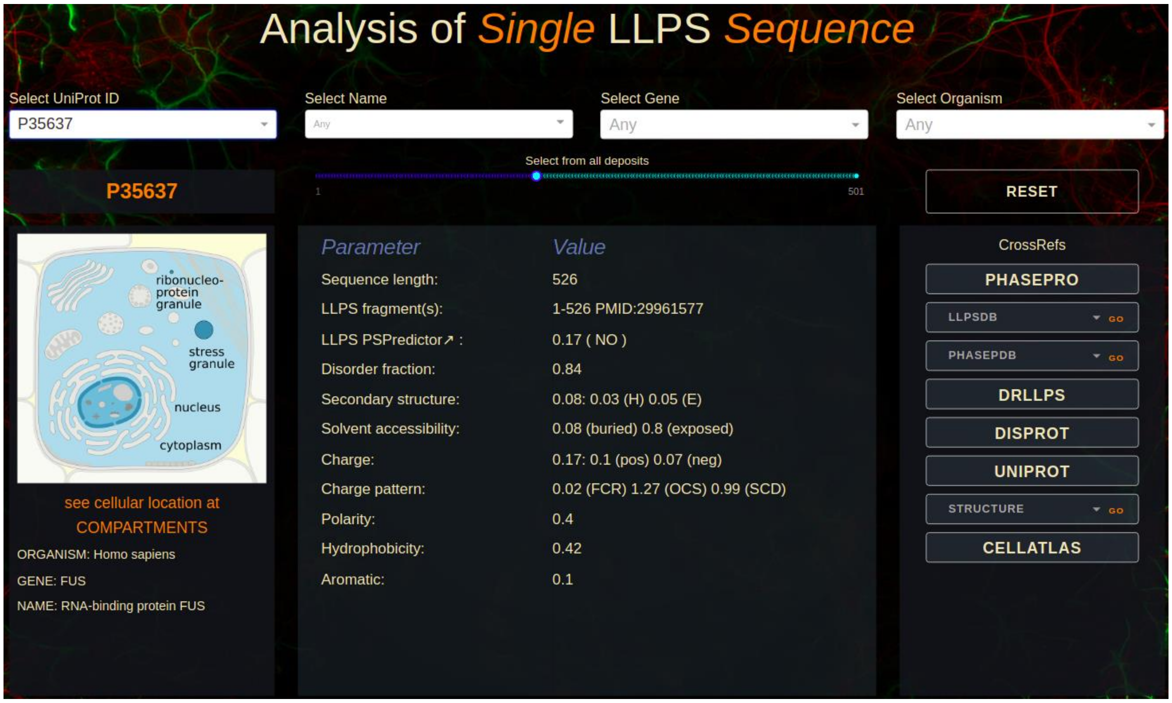

1.2 Sequence Complexity & Composition web app:

https://biapss.chem.iastate.edu/single_seq.html#complexity

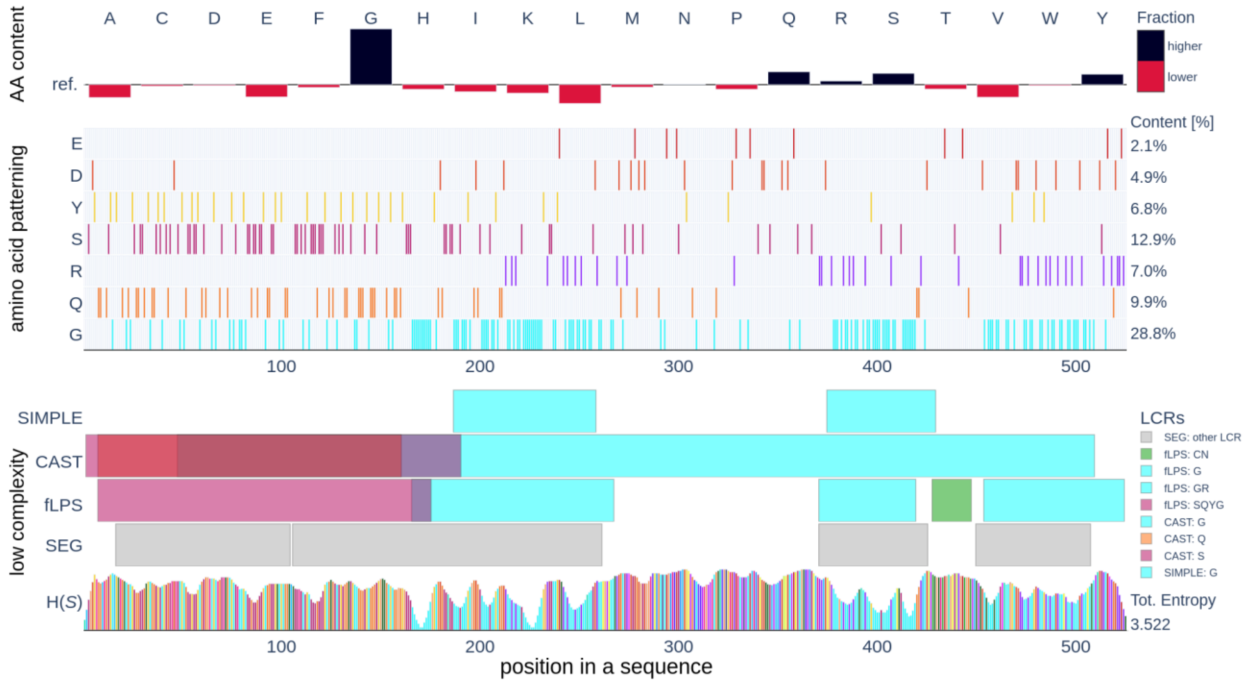

1.3 Patterns of Chemical Decoration web app: *https://biapss.chem.iastate.edu/single_seq.html#pattern*

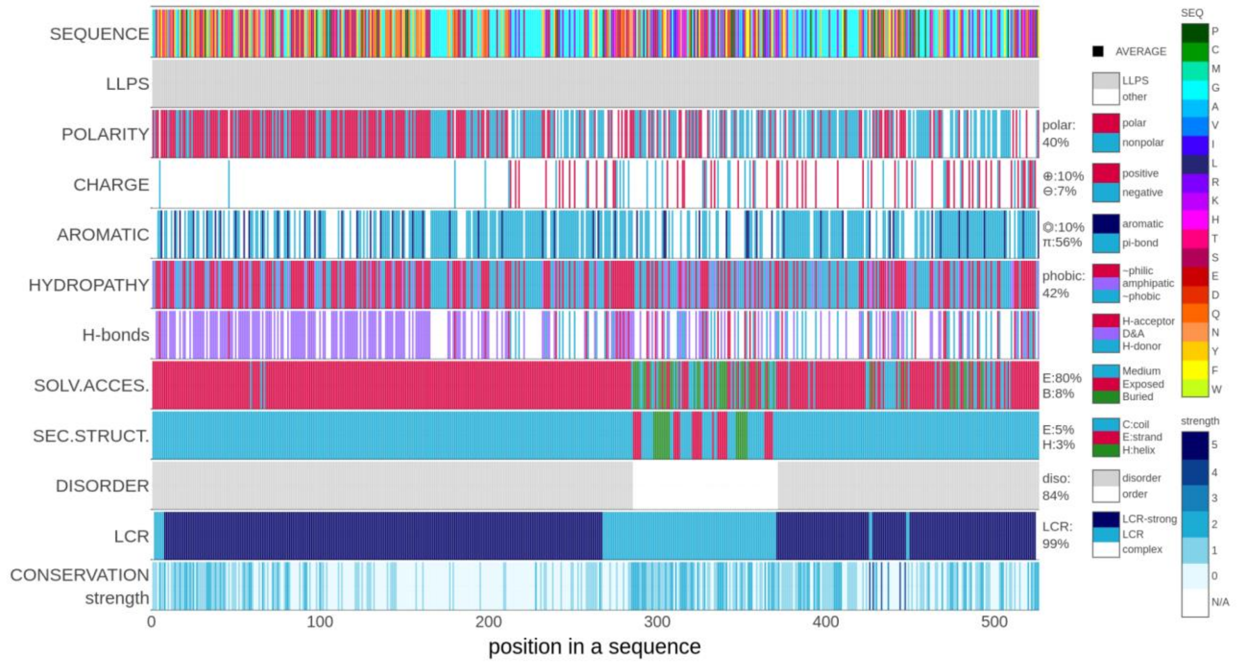

1.4 Domains, Motifs, Repeats web app: [*https://biapss.chem.iastate.edu/single_seq.html#motifs*](https://biapss.chem.iastate.edu/single_seq.html#motifs)

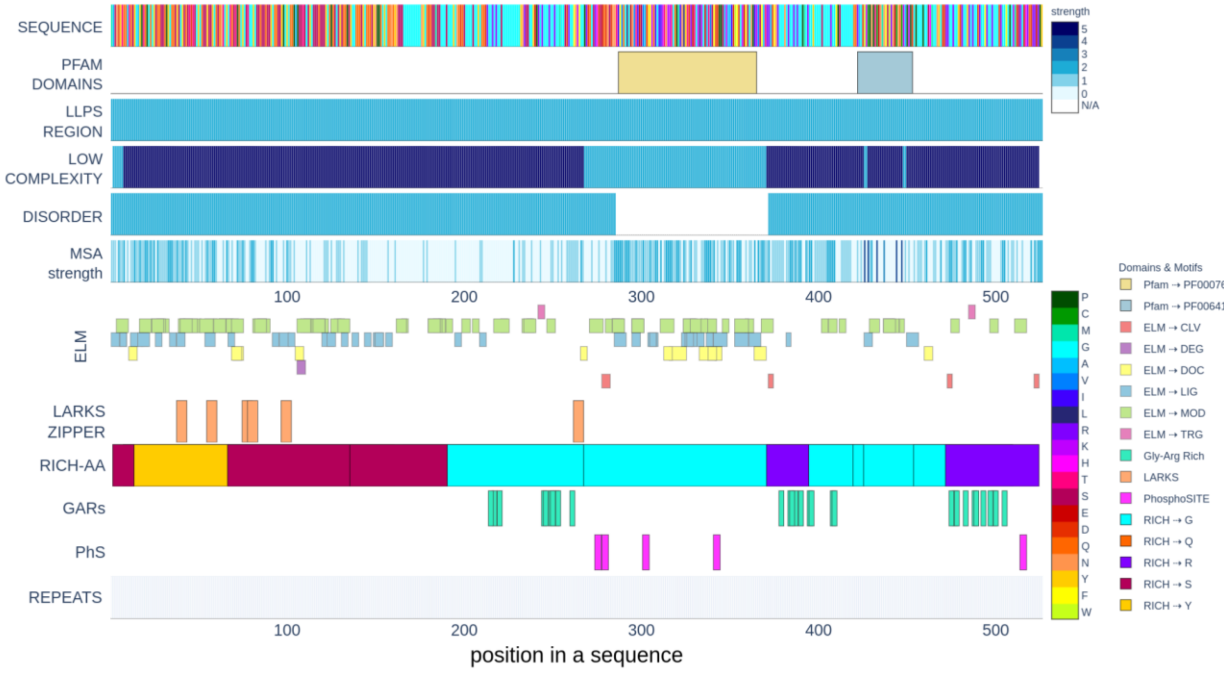

1.5 Sequence Conservation web app: [*https://biapss.chem.iastate.edu/single_seq.html#conservation*](https://biapss.chem.iastate.edu/single_seq.html#conservation)

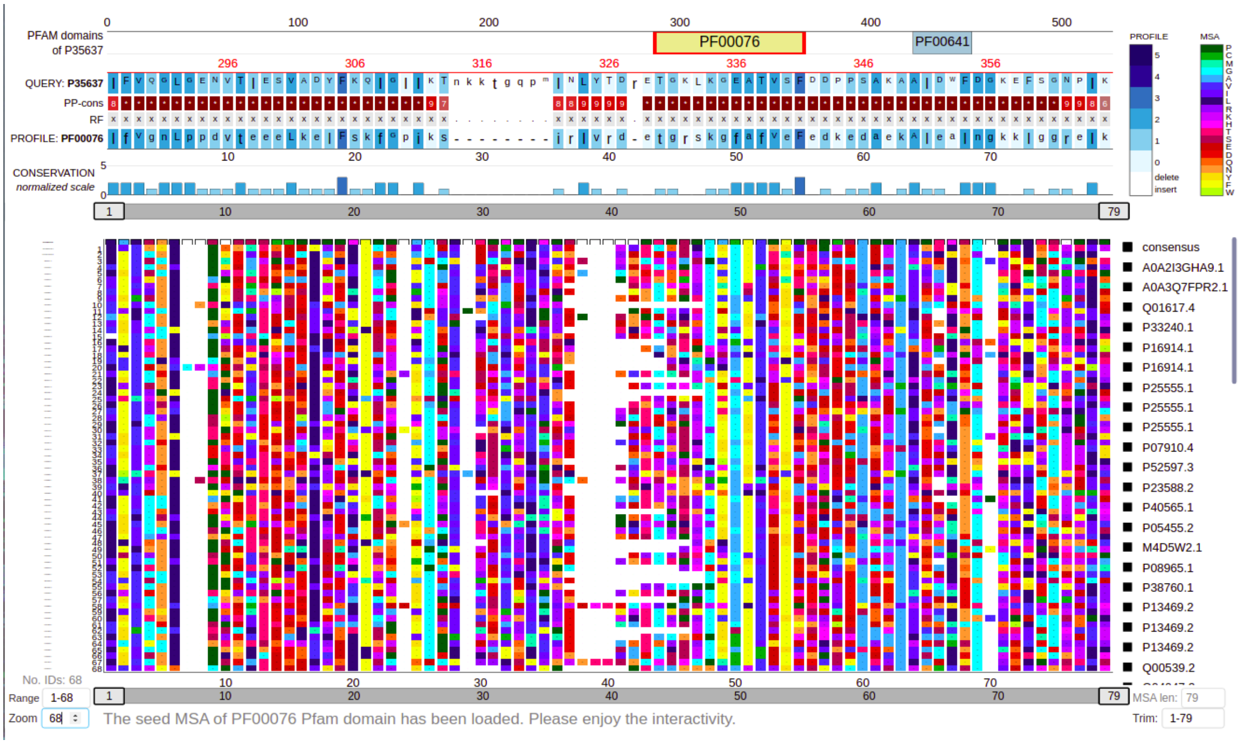

Domain PF00076

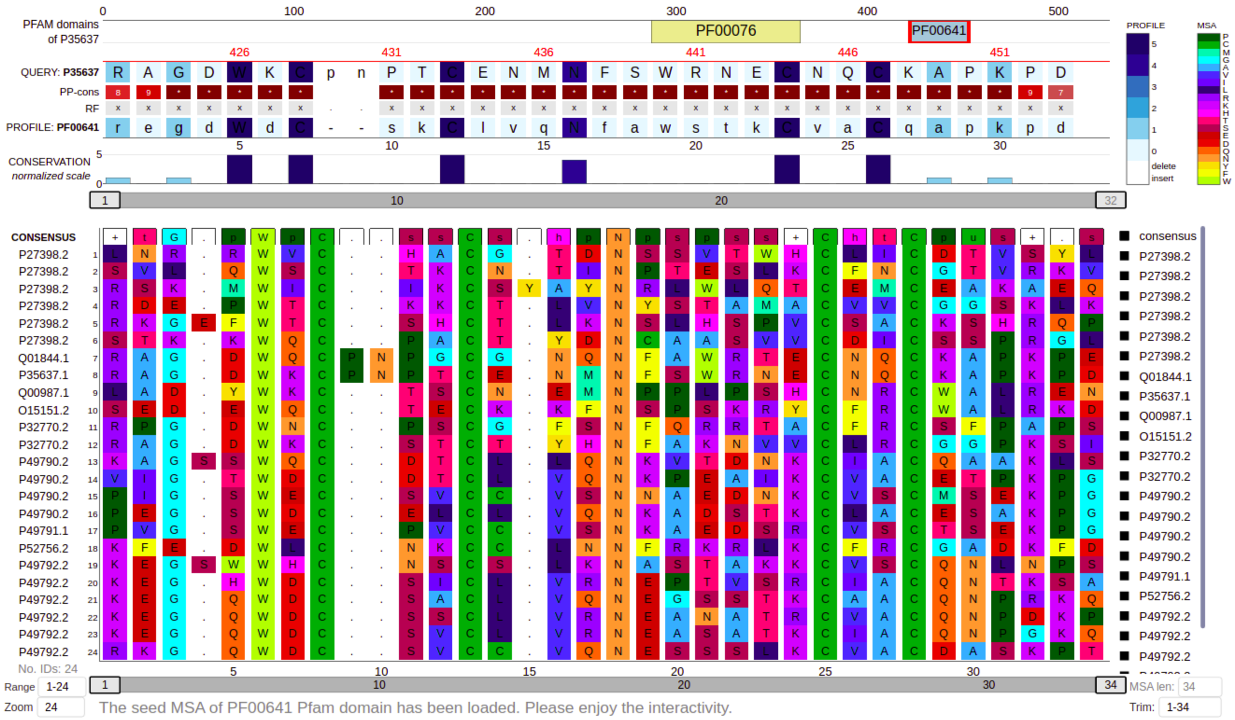

Domain PF00641

1.6 The Secondary Structure web app: [*https://biapss.chem.iastate.edu/single_seq.html#secstruct*](https://biapss.chem.iastate.edu/single_seq.html#secstruct)

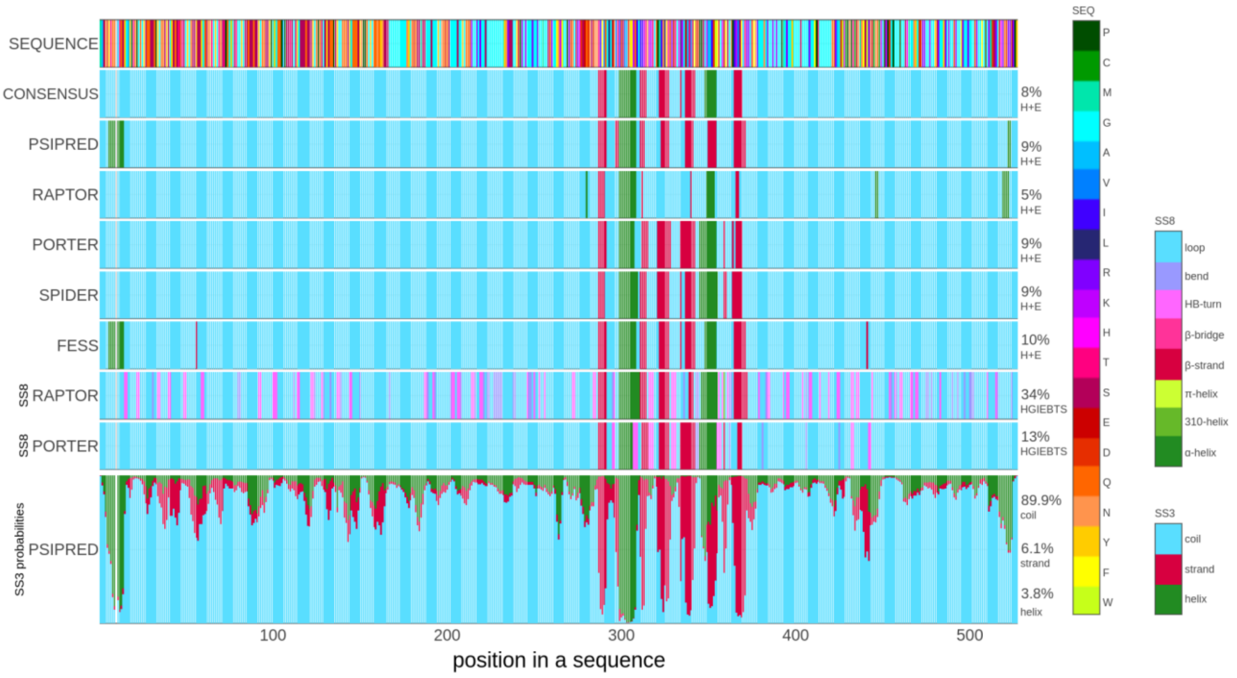

1.7 The Solvent Accessibility web app: https://biapss.chem.iastate.edu/single_seq.html#solventacc

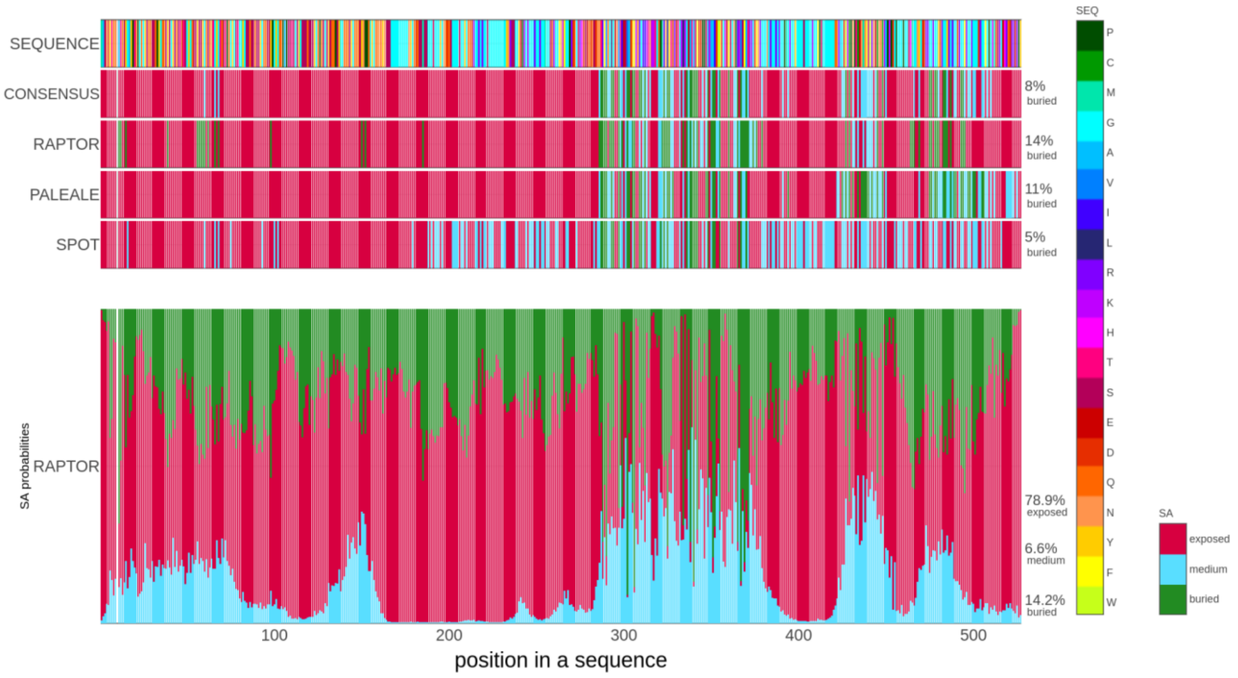

1.8 The Structural Disorder web app: https://biapss.chem.iastate.edu/single_seq.html#disorder

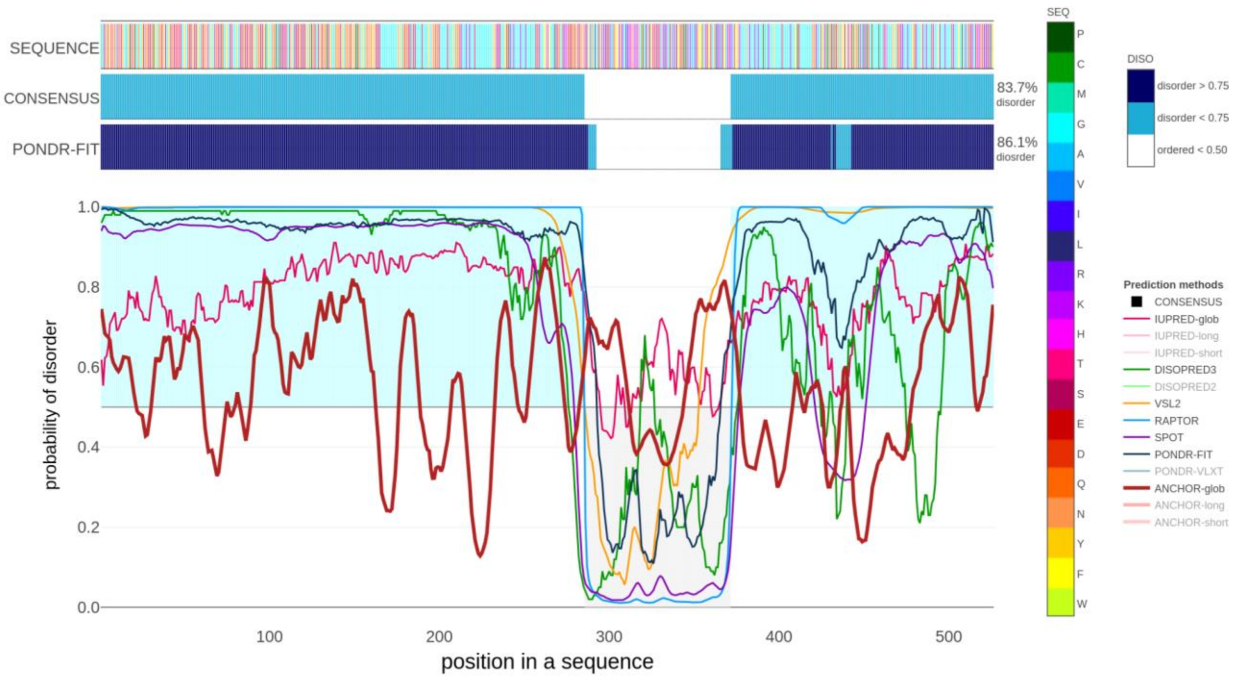

1.9 Contact Map web app: [*https://biapss.chem.iastate.edu/single_seq.html#contacts*](https://biapss.chem.iastate.edu/single_seq.html#contacts)

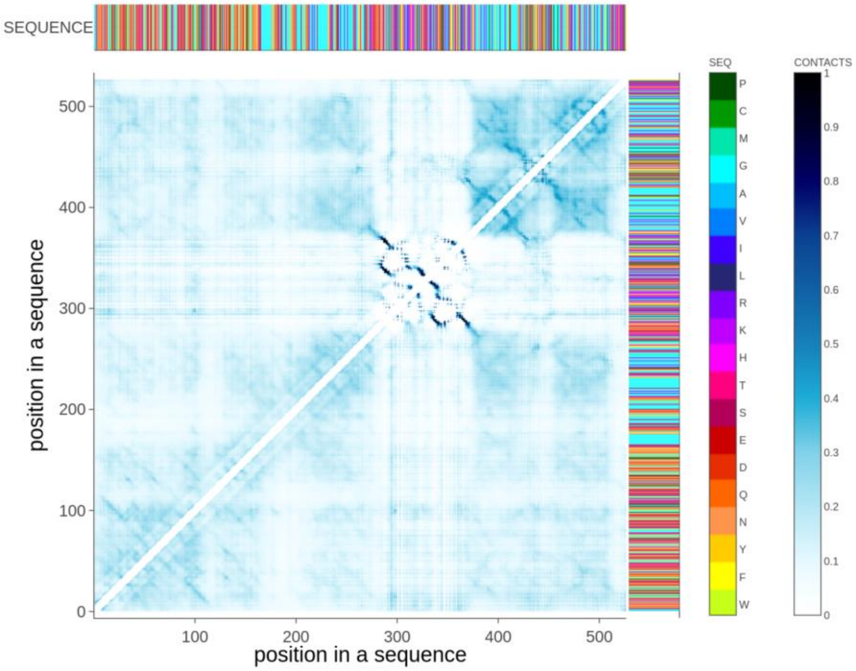

Supplement: Supplementary file 1 [file ijms-23-06204-s001.zip › ijms-1730156-supplementary.pdf]
